# Network and Pairwise Meta‐Analysis of the Association Between Novel Hypoglycemic Agents and Atrial Fibrillation Risk in Patients With Type 2 Diabetes Mellitus

**DOI:** 10.1002/dmrr.70202

**Published:** 2026-07-15

**Authors:** Xuejiao Ye, Qian Wu, Linye Hou, Xinzheng Hou, Yingtian Yang, Chenyan Yang, Mingyu Huang, Shihan Wang

**Affiliations:** ^1^ China Academy of Chinese Medical Sciences Guang’Anmen Hospital Beijing China; ^2^ Beijing University of Chinese Medicine Beijing China

**Keywords:** atrial fibrillation, DPP‐4 inhibitors, GLP‐1 receptor agonist, network meta‐analysis, SGLT‐2 inhibitors, type 2 diabetes mellitus

## Abstract

**Objective:**

To evaluate the atrial fibrillation (AF) risk of sodium‐glucose cotransporter‐2 inhibitors (SGLT‐2i), glucagonlike peptide‐1 receptor agonists (GLP‐1RA) and dipeptidyl peptidase‐4 inhibitors (DPP‐4i) in patients with Type 2 Diabetes Mellitus (T2DM) with network meta‐analysis.

**Methods:**

Systematic literature searches were conducted of MEDLINE, Cochrane Central Register of Controlled Trials (CENTRAL), Embase, and Clinical Trials.gov covering inception till January 31, 2026. Randomized control trials (RCTs) and cohort studies comparing SGLT‐2i, GLP‐1RA and DPP‐4i in diabetes were selected. We performed a network meta‐analysis to compare the three drugs indirectly. Results were reported as risk ration (RR) with corresponding 95% confidence interval (CI).

**Results:**

4 RCTs and 21 cohort studies involving 2,171,267 patients were included. Compared with GLP‐1RA [RR with 95% CI: 0.86(0.79, 0.95), RR with 95% CI: 0.87(0.78, 0.97)] and DPP‐4i[RR with 95% CI: 0.80(0.74, 0.87), RR with 95% CI: 0.81(0.74, 0.88)], SGLT‐2i significantly reduced the risk of AF and new‐onset AF. While there were no significant difference in the risk of AF recurrence among SGLT‐2i, GLP‐1RA, and DPP‐4i[RR with 95% CI: 0.87(0.68, 1.12), RR with 95% CI: 0.80(0.61, 1.05), RR with 95% CI: 0.92(0.63, 1.33)]. There were also no significant difference in the risk of AF and new‐onset AF between GLP‐1RA and DPP‐4i[RR with 95% CI: 0.93(0.84, 1.03), RR with 95% CI: 0.93(0.83, 1.04)].

**Conclusions:**

The management of T2DM involves the prevention of subsequent cardiovascular complications. The results of this network meta‐analysis indicate that SGLT2i is associated with a lower risk of AF in T2DM patients compared to GLP‐1RA and DPP‐4i. Sensitivity analysis further confirms that SGLT‐2i significantly reduces the risk of AF recurrence statistically. These finding suggests that SGLT‐2i should be considered as a preferred antidiabetic regimen for patients with T2DM at high risk of AF.

## 1 Introduction

As the global population ages and dietary patterns change, the prevalence of diabetes mellitus (DM) has been on a continuous upward trend, emerging as a major public health challenge worldwide. By 2021, there were 536.6 million individuals had been diagnosed with DM, and this number is projected to reach 643 million by 2030, and exceed 783.2 million by 2045. In addition, approximately 541 million individuals suffered from impaired glucose tolerance. Direct medical expenditures related to diabetes have approached 1 trillion USD, and it is expected to exceed this threshold by 2030 [1]. Poor control of diabetes and its complications not only severely impairs patients' quality of life, but also poses a significant threat to their survival, further burdening the global healthcare system.

As clinical research continues to deepen, the treatment of type 2 DM (T2DM) should not be limited to simple blood glucose control, but also give priority to the prevention and management of cardiovascular complications. Atrial fibrillation (AF) is one of the most common cardiac arrhythmias in clinical practice, and DM has been confirmed as an independent risk factor for it. Moreover, due to shared risk factors such as obesity, hypertension, and advanced age, these two diseases often coexist [2, 3]. The Framingham Heart Study (FSH) was the first to demonstrate that DM significantly increases the risk of AF [4]. Subsequent studies further quantified this association, clearly indicating that T2DM can increase the relative risk of AF by 35%–60% [4, 5]. The incidence of AF in diabetic patients over 65 years old can reach 27% within a decade [6]. Cumulative exposure to diabetes is also associated with an increased risk of AF. Each additional year of DM duration increases the risk of AF by 3% [7]. More importantly, T2DM combined with newly diagnosed AF can significantly worse patient prognosis. Compared with patients with T2DM alone, those with T2DM complicated by new‐onset AF have a 3.8 fold higher risk of heart failure (HF) and a 2.7 fold higher risk of all‐cause mortality [8]. Furthermore, patients with AF complicated by diabetes have a more severe symptom burden and higher cardiovascular and cerebrovascular mortality [9, 10]. Once AF occurs, it forms a vicious cycle of atrial electrophysiological and structural remodeling, leading to further deterioration of clinical outcomes. Thus, upstream treatment is particularly crucial for preventing AF in patient with T2DM.

Despite traditional hypoglycemic agents can effectively control blood glucose, their cardiovascular protective benefits are limited [11]. In contrast, novel glucose‐lowering agents such as sodium glucose cotransporter‐2 inhibitors (SGLT‐2i) [12, 13], glucagon‐like peptide‐1 receptor agonists (GLP‐1RA) [14], and dipeptidyl peptidase‐4 inhibitors (DPP‐4i) [15, 16] not only demonstrate significant glucose‐lowering efficacy, but also have been proven to exert definite cardiovascular protective effects. They can effectively reduce the incidence of major adverse cardiovascular events (MACEs) and the risk of hospitalization for HF [17]. Current evidence suggests that the mechanism by which diabetes induces AF may be related to atrial structural remodeling, electrical remodeling, and autonomic nervous system remodeling, with hyperglycemia and elevated glycosylated hemoglobin (HbA1c) levels being important driving factors. Rational glycemic control can reduce the risk of AF and improve long‐term prognosis in DM patients [18].

Network meta‐analysis, which integrates evidence from direct and indirect comparisons and achieves comprehensive efficacy evaluation through algorithmic optimization, is an efficient research method to assist clinical decision making. Even in the absence of head‐to‐head trials, it can still generate ranking results of different interventions. Several previously published meta‐analysis have explored the relathionship between hypoglycemic drugs and the risk of AF in patients with T2DM. However, most studies only focus on new AF events and do not comprehensively evaluate the composite outcome of AF [19]. At the same time, there are few existing meta‐analysis that include both randomized controlled trials (RCTs) and large sample cohort studies, and no standardized GRADE system has been used to grade the quality of evidence [20]. Based on this, this present study aims to systematically investigate the effectiveness of novel hypoglycemic drugs, including SGLT‐2i, GLP‐1RA, and DPP‐4i, on AF‐related outcomes in T2DM patients through a network meta‐analysis that includes RCTs and cohort studies, thereby providing evidence‐based support for optimizing clinical glucose‐lowering drug selection and improving patient prognosis.

## 2 Methods and Analysis

### 2.1 Study Registering and Reporting

This systematic review and meta‐analysis was conducted and reported in conformity with the Cochrane and Preferred Reporting Items for Systematic Review and Meta‐Analyses (PRISMA) guidelines [21]. We prospectively registered this systematic review in the International Prospective Register of Systematic Reviews database (PROSPERO) (registration number: CRD420261287440).

### 2.2 Literature Search

The search strategy was performed in accordance with the PRISMA extension statement for network meta‐analysis. To identify eligible trials, an electronic search of MEDLINE, Cochrane Central Register of Controlled Trials (CENTRAL), Embase, and Clinical Trials.gov was conducted from inception till January 30, 2026. No time or language restrictions were set. Meanwhile, the reference lists of included studies, previous relevant meta‐analyses, and review articles were manually screened to further identify potentially eligible studies, thereby minimizing the risk of missing relevant literature. The search strategy utilized terms such as “SGLT‐2 inhibitors”, “SGLT‐2i”, “DDP‐4 inhibitors”, “DPP‐4i”, “GLP‐1 receptor agonist”, “GLP‐1RA”, “atrial fibrillation”, “AF”, “Randomized controlled trials” and “cohort study” along with their respective abbreviations and synonymous variants. The literature screening process was independently conducted by two blinded investigator (X.J. Ye and Q. Wu), who screened all retrieved literature one by one in strict accordance with the predefined inclusion and exclusion criteria. When the two authors encountered the inconsistencies, a third author (S.H. Wang) was consulted to reach a decision. The detailed search strategy for each database is presented in Table 1 and Table S4. System retrieval strategy for MEDLINE, Table S5. System retrieval strategy for CENTRAL, Table S6. System retrieval strategy for Embase, Table S7. System retrieval strategy for Clinical Trials.gov.

**TABLE 1:** System retrieval strategy.

#1 Sodium‐Glucose Transporter 2 Inhibitors [MeSH Terms]
#2 Sodium‐Glucose Transporter 2 Inhibitors [Title/Abstract]
#3 SGLT‐2 inhibitors [MeSH Terms]
#4 SGLT‐2i [Title/Abstract]
#5 #1 OR #2 OR #3 OR #4
#6 Dipeptidyl‐Peptidase IV Inhibitors [MeSH Terms]
#7 Dipeptidyl‐Peptidase IV Inhibitors [Title/Abstract]
#8 DPP‐4 inhibitors [MeSH Terms]
#9 DPP‐4i [Title/Abstract]
#10 #6 OR #7 OR #8 OR #9
#11 Glucagon‐Like Peptide‐1 Receptor Agonists [MeSH Terms]
#12 Glucagon‐Like Peptide‐1 Receptor Agonists [Title/Abstract]
#13 GLP‐1 receptor agonist [MeSH Terms]
#14 GLP‐1RA [Title/Abstract]
#15 #11 OR #12 OR #13OR #14
#16 Atrial Fibrillation [MeSH Terms]
#17 atrial fibrillation [Title/Abstract]
#18 AF [Title/Abstract]
#19 #16 OR #17 OR #18
#20 Randomized controlled Trials [MeSH Terms]
#21 Randomized controlled trials [Title/Abstract]
#22 RCTs [Title/Abstract]
#23 #20 OR #21 OR #22
#24 #5 OR #10 OR #15 AND #19 AND #23

### 2.3 Inclusion Criteria

Studies were selected if they met the following criteria: (i) they were published in peer‐reviewed journal; (ii) they included adult patients (≥ 18 years old) with T2DM with or without AF; (iii) any RCTs or cohort studies that reported AF outcomes in patients treated with SGLT‐2i, DPP‐4i or GLP‐1RA, compared with placebo, standard care, or other active antidiabetic agents (including head‐to‐head comparisons between the three drug classes). The exclusion criteria were as follows: (i) duplicate papers correlated with same trial; (ii) non‐clinical trials, systematic reviews, case reports, editorials, and others; (iii) studies failed to report the outcomes of interest.

### 2.4 Data Extraction

Two researchers (X.J. Ye and Q. Wu) used EndNote X9 for reference management, removing duplicate records and excluding irrelevant or non‐compliant studies according to the inclusion and exclusion criteria. Subsequently, a database was established using Microsoft Excel to record detailed study information, including publication details (title, first author name, and publication year), patient information (sample size, mean age, and sex), interventions (medication, dosage, administration time, follow‐up time), outcomes (the risk of AF, including recurrent AF and new onset AF), and study design (randomization, allocation concealment, and blinding). To ensure the accuracy of the data, two independent researchers entered the data and cross‐checked for inconsistencies. For studies with missing data, we contacted the original authors for clarification; if no accurate data was provided, the study will be excluded. Any disagreements between the initial reviewers will be resolved by the third reviewer, S.H. Wang.

### 2.5 Quality Assessment and Risk‐of‐Bias Evaluation

We evaluated the certainty of evidence for primary outcomes using the Grading of Recommendations Assessment, Development and Evaluation (GRADE) system. Since the present network meta‐analysis incorporated both RCTs and cohort studies, observational evidence was prone to confounding and selection bias. Accordingly, we downgraded the certainty of evidence by one level within the study‐design domain following GRADE guidance.

Differentiated risk‐of‐bias assessment tools were adopted according to study design: the Revised Cochrane Risk of Bias Tool (RoB 2) [17] for RCTs and the Risk of Bias in Non‐randomized Studies of Interventions tool (ROBINS‐I) tool for cohort studies. These two tools were applied independently, with no mathematical combination or uniform weighting of bias ratings, and assessment outcomes were presented separately. A random‐effects model was used to address heterogeneity derived from mixed study designs, and sensitivity and subgroup analyses were performed to validate the robustness of network meta‐analysis findings. Conducting network meta‐analysis combining RCTs and cohort studies complied with the PRISMA‐NMA statement and Cochrane methodological standards [22, 23].

Risk‐of‐bias evaluation was independently performed by researchers (L.Y. Hou, X.Z. Hou and Y.T. Yang) following standardized criteria and predefined domains tailored to each study type to guarantee objectivity and consistency. For RCTs, RoB 2 was used, which includes bias arising from the randomization process, bias due to deviations from intended interventions, bias due to missing outcome data, bias in outcome measurement, and bias in selection of the reported result. The study will be classified as overall low bias risk only when all five domains are estimated as low risk. An RCT was rated as overall low risk of bias only if all five domains were judged low‐risk. For cohort studies, ROBINS‐I was used, which is a standard bias assessment tool recommended by the Cochrane Collaboration for non‐randomized intervention studies. It includes seven predefined core domains, classified by the intervention implementation stage as follows: pre‐intervention (bias due to confounding, bias in selection of participants into the study), intra‐intervention (bias in classification of interventions), and post‐intervention (bias due to deviations from intended interventions, bias due to missing data, bias in measurement of outcomes, bias in selection of the reported result). A cohort study will be classified as overall low bias risk only when all seven domains are estimated as low risk. Any discrepancies are resolved through discussion; if necessary, third authors (S.H. Wang) with greater expertise can be involved.

### 2.6 Statistical Analyses

We performed this network meta‐analysis using Stata 14.0 and R 4.4.3 statistical software. Stata 14.0 software mainly completes core statistical analysis steps such as data organization, network graph drawing, heterogeneity testing, meta regression analysis, and node splitting method inconsistency testing. The R 4.4.3 software uses the “gemtc” and “rjag” packages to complete the core calculations of network meta‐analysis and generate league tables. In addition, we use GraphPad Prism software to draw forest plots, ensuring clear and standardized visualization of statistical results, making it easy for readers to quickly understand and intuitively interpret research results. Risk ration (RR) and 95% confidence interval (CI) were used to present the efficacy of treatments. For dichotomous outcomes, results were presented as RR with corresponding 95% CI. For continuous variable outcomes, results were shown as mean difference (MD) with 95% CI. Heterogeneity among included studies was evaluated using the I^2^ statistic and Cochran's Q test. Heterogeneity was categorized as follows: unimportant (0% < I^2^ < 40%), moderate (30% < I^2^ < 60%), substantial (50% < I^2^ < 90%), considerable (75% < I^2^ < 100%). Sensitivity analyses was conducted to assess the robustness of the results. To explore potential effect modification and ensure more robust and accurate efficacy comparisons, pre‐specified subgroup analyses were conducted based on the type of included studies, follow‐up duration, and outcome measures. Meta‐regression analyses were undertaken to quantitatively investigate the potential linear association of AF‐related risk and relevant moderating variables. *p* < 0.05 indicates statistically significant difference.

## 3 Results

### 3.1 Transitivity Testing

This network meta‐analysis conducted a systematic transitivity test by comprehensively comparing key baseline characteristics such as sample size, follow‐up duration, and male proportion between groups. All transitivity tests had *p*‐values greater than 0.05, indicating good comparability between different intervention measures and meeting the core hypothesis of network meta‐analysis. The detailed results were shown in Table S1. Transitivity testing results for network meta‐analysis.

### 3.2 Inconsistency Testing

This study used the node splitting method and the design by treatment interaction model to evaluate the inconsistency of all network evidence loops. The results showed that there was no statistically significant inconsistency between direct and indirect evidence in all loops (all *p* > 0.05), further confirming the robustness and reliability of the network meta‐analysis results in this study. The detailed results were shown in Table S2. Inconsistency assessment by node splitting method.

### 3.3 Literature Search and Included Studies

**FIGURE 1:**
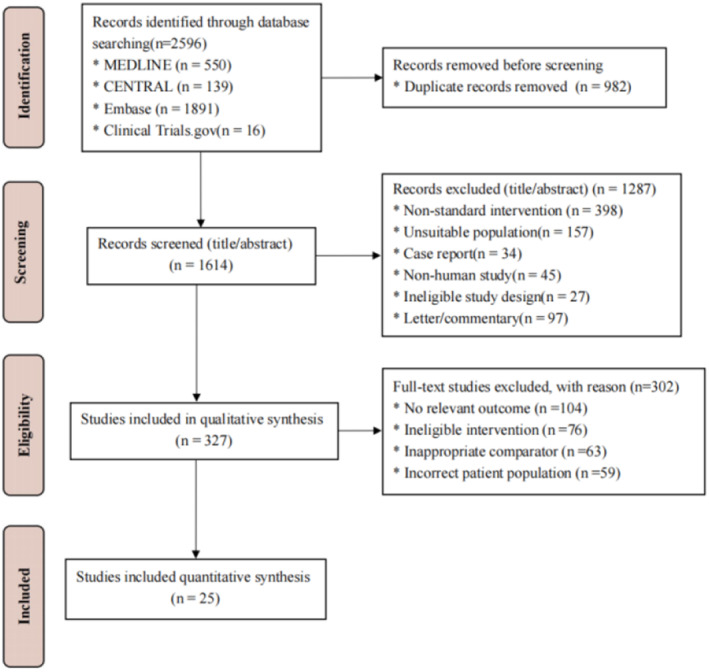
PRISMA flowchart of study selection process.

The detailed study filtering process was shown in Figure 1. In brief, we retrieved a total of 2596 articles from MEDLINE (*n* = 550), Cochrane Central Register of Controlled Trials (CENTRAL) (*n* = 139), Embase (*n* = 1891), and Clinical Trials.gov (*n* = 16). A total of 982 duplicate articles were removed. After review by title and abstract, 1287 articles were removed due to: Non‐standard intervention (*n* = 398), unsuitable population (*n* = 157), case report (*n* = 34), non‐human (*n* = 45), design (*n* = 27), letter or commentary or abstract (*n* = 97), review or meta‐analysis (*n* = 529). After that, 268 articles remained and entered into full‐text assessing section. By assessing full text, 243 additional articles were excluded due to the lack of relevant outcome indicators. Finally, 25 articles were included in this network meta‐analysis. Out of 25 studies, 8 studies were compared SGLT‐2i and GLP‐1RA, 11 studies were compared SGLT‐2i and DPP‐4i, 3 studies were compared GLP‐1RA and DPP‐4i, 2 studies were compared SGLT‐2i and placebo, 1 studies were compared GLP‐1RA and placebo.

### 3.4 Baseline Characteristics of Included Studies

Baseline characteristics of included studies are summarized in Table 2. The pooled population consisted of 2,171,267 patients with T2DM, 707,172 of them were in SGLT‐2i studies, 307,535 of them were in GLP‐1RA studies, 1,136,662 of them were in DPP‐4i studies, 19,898 of them were in placebo studies. There were 1,540,288 male patients, accounting for 70.9% of the total sample size. The included studies comprised 4 RCTs and 21 cohort studies. The majority of the included studies consists of two arms and was published between 2020 and 2025. The follow‐up duration of these studies ranged from 12 to 67.7 months.

**TABLE 2:** Baseline characteristics of included studies.

Study	Treatment	Number of patients		Age	Male	Follow‐up (months)	BMI (kg/m^2^)	HbA1c (%)	eGFR (mL/min/1.73 m^2^)	Design
		E	T							
Sardu C (2025) [24]	I	60	220	53.9 (8.4)	142	12	26.6 (5.95)	6.5 (0.3)	68.9 (21.3)	PCS
	C	58	193	54.5 (8.1)	119	12	25.5 (4.6)	6.7 (0.6)	70.7 (22.8)	
Kishima H (2022) [25]	I	9	38	70.3 (8.6)	26	12	N/A	N/A	N/A	RCT
	C	15	32	70.3 (7.7)	22	12	N/A	N/A	N/A	
Fichadiya A (2024) [26]	I	97	1121	64.8 (10.2)	5034	36	N/A	N/A	N/A	RCS
	C	112	1121	68 (10.5)	8578	36	N/A	N/A	N/A	
Palanca A (2024) [27]	I	45	1848	57.68 (10.74)	9520	3.17 (2.12)	N/A	N/A	N/A	RCS
	C	101	5034	61.26 (11.07)	80475	3.17 (2.12)	N/A	N/A	N/A	
Zelniker TA (2020) [28]	I	264	8582	N/A	N/A	50	N/A	N/A	N/A	RCT
	C	325	8578	N/A	N/A	50	29.9 (6.7)	8.9 (1.7)	N/A	
Yen FS (2025) [29]	I	150	9520	56.89 (11.07)	5774	60	28.05 (4.85)	8.86 (1.67)	94.28 (31.15)	RCS
	C	180	9520	57.1 (10.89)	5727	60	N/A	N/A	N/A	
Zhuo M (1) (2022) [30]	I	1175	80475	71.8 (5.1)	36025	12	N/A	N/A	N/A	RCS
	C	1235	80475	71.8 (5.1)	35977	12	N/A	N/A	N/A	
Zhuo M(2) (2022) [30]	I	1082	74868	71.8 (5)	36398	12	N/A	N/A	N/A	RCS
	C	1410	74868	71.7 (5.1)	36302	12	28.3 (5.5)	8.9 (1.8)	83.9 (32.8)	
Li YJ (2024) [31]	I	139	16487	67.09 (9.65)	2746	12	N/A	N/A	N/A	RCS
	C	947	80949	72.2 (10.14)	245442	12	N/A	N/A	N/A	
Lui DT (2023) [32]	I	15	2920	55.4 (12.4)	1630	17	N/A	N/A	N/A	RCS
	C	35	2920	55.6 (12.6)	1644	17	N/A	N/A	N/A	
Ling AW (2020) [33]	I	93	15606	58.52 (11.76)	470358	1.48 (0.81, 2.10)	36.4 (7.2)	8.1 (2)	95 (18)	RCS
	C	146	12383	62.52 (12.58)	9424	1.05 (0.60, 1.67)	37.7 (7.6)	7.8 (1.9)	94 (18)	
Lee S (2023) [34]	I	426	21713	57.6 (11.3)	13011	67.7	N/A	N/A	N/A	RCS
	C	604	21713	59.1 (11.2)	12995	67.7	32.3 (5.7)	7.3 (1.1)	77.5 (22.7)	
Li C (2022) [35]	I	193	7997	63 (8.5)	N/A	30	34.3 (6.2)	8.5 (1.4)	N/A	RCT
	C	161	6546	63 (8.5)	N/A	30	26.1 (5.62)	6.6 (0.3)	70.8 (20.8)	
Lee J (2023) [36]	I	147	42786	N/A	N/A	15.6	25.3 (4.3)	6.8 (0.9)	76.1 (21.1)	RCS
	C	176	42786	N/A	N/A	15.6	N/A	N/A	N/A	
Kim M (2024) [37]	I	86	42786	54.7 (12.1)	N/A	15.6	N/A	N/A	N/A	RCS
	C	108	42786	54.7 (12.2)	N/A	15.6	N/A	N/A	N/A	
Hsiao FC (2022) [38]	I	51	16566	58.5 (12)	9526	1.52 (0.74)	N/A	N/A	N/A	RCS
	C	11	2746	58.2 (12.3)	1532	1.33 (1.12)	N/A	N/A	N/A	
Chan YH(1) (2022) [39]	I	1518	245442	59 (12.8)	138611	2.02 (1.3)	N/A	N/A	N/A	RCS
	C	1671	245442	60.3 (11.7)	138350	2.02 (1.3)	N/A	N/A	N/A	
Chan YH(2) (2022) [39]	I	228	43682	56.8 (11.9)	22333	2.2 (1.3)	30.4 (6.5)	8.9 (1.7)	N/A	RCS
	C	305	43682	55.2 (13.2)	22517	2.2 (1.3)	26.29 (5.89)	8.37 (1.97)	77.07 (41.83)	
Chan YH(3) (2022) [39]	I	289	39190	55.4 (13.5)	20440	2.3 (1.3)	N/A	N/A	N/A	RCS
	C	282	39190	58.6 (11.3)	20355	2.3 (1.3)	N/A	N/A	N/A	
Park S (2023) [40]	I	147	44557	N/A	N/A	15.6	N/A	N/A	N/A	RCS
	C	1270	470358	N/A	N/A	15.6	N/A	N/A	N/A	
Xu YW(1) (2025) [41]	I	150	9424	53 (11)	N/A	46	28.5 (5.2)	9 (1.6)	87.6 (32)	RCS
	C	135	9424	53 (11)	N/A	46	N/A	N/A	N/A	
Xu YW(2) (2025) [41]	I	285	14566	52 (11)	N/A	46	N/A	N/A	N/A	RCS
	C	337	14566	52 (11)	N/A	46	N/A	N/A	N/A	
Shin JI (2023) [42]	I	956	26774	N/A	N/A	51.6	N/A	N/A	N/A	RCS
	C	619	17348	N/A	N/A	51.6	36.8 (7.7)	8.1 (1.9)	95 (18)	
Peter JR (2021) [43]	I	269	4769	66.1 (6.5)	N/A	64.8	37.9 (8.4)	7.9 (1.9)	94 (20)	RCT
	C	255	4774	66.1 (6.5)	N/A	64.8	N/A	N/A	N/A	
Khalil S (2025) [44]	I	3319	80948	64 (11)	74621	32	32.3 (5.7)	7.3 (1.1)	76.8 (22.8)	RCS
	C	3481	80948	64 (11)	74634	32	34.4 (6.4)	8.4 (1.4)	N/A	

Abbreviations: C, control group; I, intervention group; N/A, not available; PCS, prospective cohort study; RCS, retrospective cohort study; RCT, randomized controlled trial.

### 3.5 Risk of Bias

**FIGURE 2:**
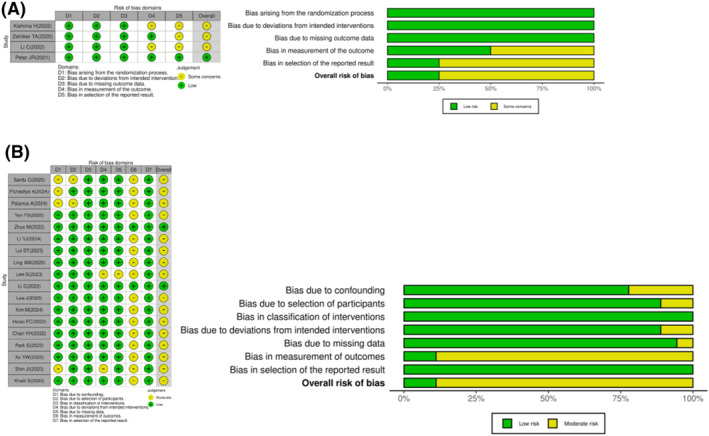
(A) Risk of bias summary of included RCTs. (B) Risk of bias summary of included cohort studies.

For the included RCTs, we assessed the risk of bias using the Revised Cocharane Risk of Bias Tool (RoB 2.0), with results presented in Figure 2A. Except for Kishina H . (2022), Zelniker T. A . (2020) and Li C . (2022), all trials were evaluated as low risk in the 5 core domains. For the included cohort studies, we assessed the risk of bias using the RoBINS‐I tool, with results shown in Figure 2B. Only two studies, Zhuo M. (2022) and Li C . (2022), were evaluated as low risk of bias across the core 7 domains. All other studies were evaluated as moderate risk of bias. Risk of bias assessments for RCTs and cohort studies are summarized in Figure 2A,B, respectively.

### 3.6 GRADE Quality Assessment

In terms of reducing the risk of AF, there were 25 direct comparisons in the original articles, of which 3 studies were estimated high quality, 14 studies were estimated moderate quality, 8 studies were estimated low quality. In terms of reducing the risk of AF recurrence, there were 5 direct comparisons in the original articles, with 1 study was estimated high quality, 2 studies were estimated moderate quality, and 2 studies were estimated low quality. In terms of incident of new‐onset AF, there were 20 direct comparisons in the original articles. Among them, 2 study were estimated high quality, 18 studies were estimated moderate quality. GRADE quality assessment results are summarized in Table 3.

**TABLE 3:** GRADE assessment.

Certainty assessment							No. of patients		Certainty	Importance
Intervention of studies	No. of studies	Study design	Risk of bias	Inconsistency	Indirectness	Imprecision	Intervention group	Control group		
Total AF risk										
SGLT‐2i vs. GLP‐1RA	8	PCS + RCS	Serious	Not serious	Not serious	Serious	2384/175669 (1.4%)	2795/168062 (1.7%)	⨁⨁◯◯ Low	IMPORTANT
SGLT‐2i vs. DPP‐4i	11	RCT + RCS	Serious	Not serious	Not serious	Not serious	3894/514924 (0.8%)	6639/1001958 (0.7%)	⨁⨁⨁◯ Moderate	IMPORTANT
SGLT‐2i vs. placebo	2	RCT	Not serious	Not serious	Not serious	Not serious	457/16579 (2.8%)	486/15124 (3.2%)	⨁⨁⨁⨁ High	IMPORTANT
GLP‐1RA vs. DPP‐4i	3	RCS	Serious	Not serious	Not serious	Not serious	3893/134704 (2.9%)	4100/134704 (3.0%)	⨁⨁⨁◯ Moderate	IMPORTANT
GLP‐1RA vs. placebo	1	RCT	Not serious	Not serious	Not serious	Not serious	269/4769 (5.6%)	255/4774 (5.3%)	⨁⨁⨁⨁ High	IMPORTANT
Risk of AF recurrence										
SGLT‐2i vs. GLP‐1RA	2	PCS + RCS	Serious	Not serious	Not serious	Serious	161/5254 (3.1%)	103/2041 (5.1%)	⨁⨁◯◯ Low	IMPORTANT
SGLT‐2i vs. DPP‐4i	2	RCT + RCS	Not serious	Not serious	Not serious	Not serious	106/1159 (9.2%)	127/1153 (11.0%)	⨁⨁⨁◯ Moderate	IMPORTANT
SGLT‐2i vs. placebo	1	RCT	Not serious	Not serious	Not serious	Not serious	264/8582 (3.1%)	325/8578 (3.8%)	⨁⨁⨁⨁ High	IMPORTANT
Risk of new‐onset AF										
SGLT‐2i vs. GLP‐1RA	6	RCS	Serious	Serious	Not serious	Not serious	2223/170415 (1.3%)	2692/166021 (1.6%)	⨁⨁⨁◯ Moderate	IMPORTANT
SGLT‐2i vs. DPP‐4i	9	RCS	Serious	Not serious	Not serious	Not serious	3788/513765 (0.7%)	6512/1000805 (0.7%)	⨁⨁⨁◯ Moderate	IMPORTANT
SGLT‐2i vs. placebo	1	RCT	Not serious	Not serious	Not serious	Not serious	193/7997 (2.4%)	161/6546 (2.5%)	⨁⨁⨁⨁ High	IMPORTANT
GLP‐1RA vs. DPP‐4i	3	RCS	Serious	Not serious	Not serious	Not serious	3893/134704 (2.9%)	4100/134704 (3.0%)	⨁⨁⨁◯ Moderate	IMPORTANT
GLP‐1RA vs. placebo	1	RCT	Not serious	Not serious	Not serious	Not serious	269/4769 (5.6%)	255/4774 (5.3%)	⨁⨁⨁⨁ High	IMPORTANT

### 3.7 GRADE Quality Assessment of Comparative Effects on AF Risk

Table 4 summarizes the GRADE quality assessment for the association between glucose‐lowering medications and AF, including total AF risk, AF recurrence, and incident new‐onset AF. For total AF risk, direct evidence for SGLT‐2i versus placebo and GLP‐1RA versus placebo was estimated as high quality, reflecting robust study design and precision in effect estimates. By contrast, DPP‐4i versus placebo only had indirect evidence available, which was downgraded to moderate quality. Direct comparisons showed SGLT‐2i versus GLP‐1RA as low quality, SGLT‐2i versus DPP‐4i and GLP‐1RA versus DPP‐4i as moderate quality. Network meta‐analysis integrating direct and indirect evidence produced consistent results. In terms of AF recurrence, direct evidence for SGLT‐2i versus placebo was estimated high quality, while GLP‐1RA versus placebo and DPP‐4i versus placebo only had indirect evidence, estimated as low and moderate quality, respectively. SGLT‐2i versus GLP‐1RA and SGLT‐2i versus DPP‐4i, also showed alignment between direct and network meta‐analysis‐derived estimates. For incident new‐onset AF, high‐quality direct evidence was estimated for SGLT‐2i versus placebo and GLP‐1RA versus placebo. DPP‐4i versus placebo again relied on indirect evidence (RR = 1.17, 95% CI: 0.93–1.48; moderate quality). Direct comparisons between SGLT‐2i versus GLP‐1RA (moderate quality), SGLT‐2i versus DPP‐4i (moderate quality), and GLP‐1RA versus DPP‐4i (moderate quality), were consistent with network meta‐analysis results, with no further downgrading of evidence quality in the pooled analysis.

**TABLE 4:** Estimates of effects and quality ratings for comparison of drugs to AF risk.

Comparison	Direct evidence		Indirect evidence		Network meta‐analysis	
	RR (95% CI)	Quality of evidence	RR (95% CI)	Quality of evidence	RR (95% CI)	Quality of evidence
Total AF risk						
SGLT‐2i vs. placebo	0.88 (0.72, 1.09)	⨁⨁⨁⨁ High	Not estimable^a^	—	0.89 (0.75, 1.06)	⨁⨁⨁⨁ High
GLP‐1RA vs. placebo	1.06 (0.8, 1.4)	⨁⨁⨁⨁ High	Not estimable^a^	—	1.03 (0.87, 1.24)	⨁⨁⨁⨁ High
DPP‐4i vs. placebo	—	—	1.11 (0.92, 1.34)	⨁⨁⨁◯ Moderate^b^	1.11 (0.92, 1.34)	⨁⨁⨁◯ Moderate
SGLT‐2i vs. GLP‐1RA	0.87 (0.78, 0.98)	⨁⨁◯◯ Low	Not estimable^a^	—	0.86 (0.79, 0.95)	⨁⨁◯◯ Low
SGLT‐2i vs. DPP‐4i	0.80 (0.73, 0.88)	⨁⨁⨁◯ Moderate	Not estimable^a^	—	0.80 (0.74, 0.87)	⨁⨁⨁◯ Moderate
GLP‐1RA vs. DPP‐4i	0.94 (0.81, 1.09)	⨁⨁⨁◯ Moderate	Not estimable^a^	—	0.93 (0.84, 1.03)	⨁⨁⨁◯ Moderate
AF recurrence						
SGLT‐2i vs. placebo	0.81 (0.65, 1.01)	⨁⨁⨁⨁ High	Not estimable^a^	—	0.81 (0.65, 1.01)	⨁⨁⨁⨁ High
GLP‐1RA vs. placebo	—	—	0.93 (0.67, 1.3)	⨁⨁◯◯ Low^b^	0.93 (0.67, 1.30)	⨁⨁◯◯ Low
DPP‐4i vs. placebo	—	—	1.02 (0.72, 1.44)	⨁⨁⨁◯ Moderate^b^	1.02 (0.72, 1.44)	⨁⨁⨁◯ Moderate
SGLT‐2i vs. GLP‐1RA	0.87 (0.68, 1.12)	⨁⨁◯◯ Low	Not estimable^a^	—	0.87 (0.68, 1.12)	⨁⨁◯◯ Low
SGLT‐2i vs. DPP‐4i	0.80 (0.61, 1.05)	⨁⨁⨁◯ Moderate	Not estimable^a^	—	0.8 (0.61, 1.05)	⨁⨁⨁◯ Moderate
GLP‐1RA vs. DPP‐4i	—	—	0.92 (0.63,1.33)	⨁⨁◯◯ Low^b^	0.92 (0.63, 1.33)	⨁⨁◯◯ Low
Incident of new‐onset AF						
SGLT‐2i vs. placebo	0.98 (0.72, 1.35)	⨁⨁⨁⨁ High	Not estimable^a^	—	0.95 (0.76, 1.18)	⨁⨁⨁⨁ High
GLP‐1RA vs. placebo	1.06 (0.79, 1.41)	⨁⨁⨁⨁ High	Not estimable^a^	—	1.09 (0.87, 1.36)	⨁⨁⨁⨁ High
DPP‐4i vs. placebo	—	—	1.17 (0.93, 1.48)	⨁⨁⨁◯ Moderate^b^	1.17 (0.93, 1.48)	⨁⨁⨁◯ Moderate
SGLT‐2i vs. GLP‐1RA	0.87 (0.76, 1.00)	⨁⨁⨁◯ Moderate	Not estimable^a^	—	0.87 (0.78, 0.97)	⨁⨁⨁◯ Moderate
SGLT‐2i vs. DPP‐4i	0.80 (0.73, 0.89)	⨁⨁⨁◯ Moderate	Not estimable^a^	—	0.81 (0.74, 0.88)	⨁⨁⨁◯ Moderate
GLP‐1RA vs. DPP‐4i	0.94 (0.80, 1.10)	⨁⨁⨁◯ Moderate	Not estimable^a^	—	0.93 (0.83, 1.04)	⨁⨁⨁◯ Moderate

*Note:* Quality of evidence ratings (per GRADE framework): High: Further research is very unlikely to change our confidence in the estimate of effect; Moderate: Further research is likely to have an important impact on our confidence in the estimate of effect and may change the estimate; Low: Further research is very likely to have an important impact on our confidence in the estimate of effect and is likely to change the estimate.

^a^ Cannot be estimated because the drug was not connected in a loop in the evidence network.

^b^ Intransitivity.

### 3.8 Network Meta‐Analysis of Treatment Groups

#### 3.8.1 Total AF Risk

**FIGURE 3:**
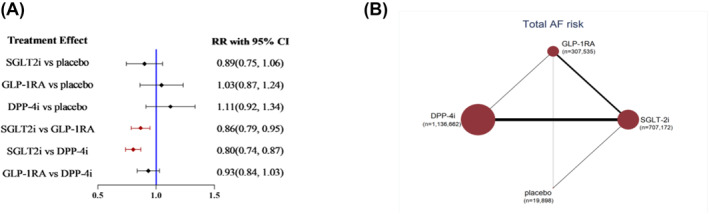
(A) Forest plots for network meta‐analyses of total AF risk. (B) Network plot for total AF risk.

Compared with GLP‐1RA[RR with 95% CI: 0.86(0.79, 0.95)] and DPP‐4i[RR with 95% CI: 0.80(0.74, 0.87)], SGLT‐2i significant decreased the risk of AF. While there was no significant difference in the risk of AF between GLP‐1RA and DPP‐4i[RR with 95% CI: 0.93(0.84, 1.03)]. There were also no significant difference in the risk of AF between SGLT‐2i[RR with 95% CI: 0.89(0.75, 1.06)], GLP‐1RA[RR with 95% CI: 1.03(0.87, 1.24)], DPP‐4i [RR with 95% CI: 1.11(0.92, 1.34)] and placebo. There was substantial heterogeneity (I^2^ = 72.6%, *p* < 0.0001). The corresponding forest plot and network plot are shown in Figure 3A,B.

#### 3.8.2 Risk of AF Recurrence

**FIGURE 4:**
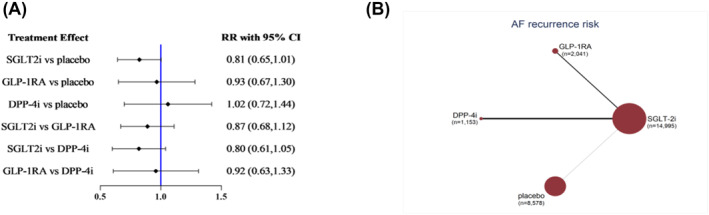
(A) Forest plots for network meta‐analyses of AF recurrence risk. (B) Network plot for AF recurrence risk.

Compared with placebo, SGLT‐2i [RR with 95% CI: 0.81(0.65, 1.01)] had a tendency to decrease the risk of AF recurrence, while GLP‐1RA [RR with 95% CI: 0.93(0.67, 1.30)] and DPP‐4i [RR with 95% CI: 1.02(0.72, 1.44)] did not. There was no significant difference among SGLT‐2i, GLP‐1RA and DPP‐4i [RR with 95% CI: 0.87(0.68, 1.12), RR with 95% CI: 0.80(0.61, 1.05), RR with 95% CI: 0.92(0.63, 1.33), respectively]in the risk of AF recurrence. This result may be related to the small sample size of the included studies. This analysis of risk of AF recurrence showed no heterogeneity (I^2^ = 12.2%, *p* = 0.3202). The corresponding forest plot and network plot are shown in Figure 4A,B.

#### 3.8.3 Risk of New‐Onset AF

**FIGURE 5:**
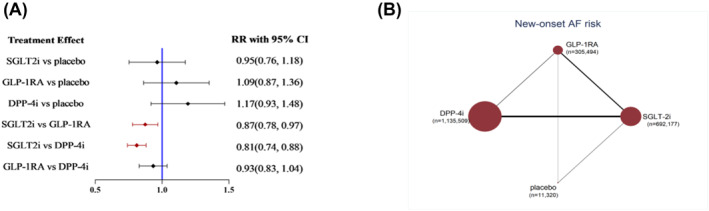
(A) Forest plots for network meta‐analyses of new‐onset AF risk. (B) Network plot for new‐onset AF risk.

SGLT‐2i significantly decreased the risk of new‐onset AF when compared with GLP‐1RA [RR with 95% CI: 0.87(0.78, 0.97)] and DPP‐4i [RR with 95% CI: 0.81(0.74, 0.88)]. GLP‐1RA didn't show a significant difference in reducing the risk of new‐onset AF compared with DPP‐4i[RR with 95% CI: 0.93(0.83, 1.04)]. There was also no significant difference in the risk of new‐onset between SGLT‐2i, GLP‐1RA, DPP‐4i and placebo [RR with 95% CI: 0.95(0.76, 1.18), RR with 95% CI: 1.09(0.87, 1.36), RR with 95% CI: 1.17(0.93, 1.48), respectively]. There was considerable heterogeneity (I^2^ = 77.2%, *p* < 0.0001). The corresponding forest plot and network plot are shown in Figure 5A,B.

### 3.9 Sensitivity Analysis

We conducted a sensitivity analysis excluding “Kishima H. (2022)”, as its small sample size, which only included seventy patients with T2DM. In terms of the risk of AF and AF recurrence, the results of sensitivity analyses were comparable to non‐exclusion of “Kishima H. (2022)”. Compared to GLP‐1RA [RR with 95% CI: 0.87(0.79, 0.95)] and DPP‐4i[RR with 95% CI: 0.81(0.75, 0.88)], SGLT‐2i significantly reduced the overall risk of AF, which consistent with the results of non‐exclusion of “Kishima H. (2022)”. In addition, compared to placebo[RR with 95% CI: 0.81(0.69, 0.95)], SGLT‐2i was associated with a decreased risk of AF recurrence. Whereas the previous results showed SGLT‐2i had a trend towards a reduction in AF recurrence compared to placebo.

We further performed Egger's regression test to assess potential publication bias and small‐study effects. The intercept‐based Egger's test indicated no significant small‐study effects (*p* = 0.156), suggesting the absence of substantial publication bias in the present analysis, which further verified the robustness of our pooled outcomes. The detailed results was shown in Supporting Information S1: Figure S1. Egger's regression test for Publication Bias.

### 3.10 Subgroup Analyses and Meta‐Regression

The subgroup analyses results showed that for recurrent AF, no significant inter‐intervention differences in efficacy were observed (all *p* > 0.05) with low heterogeneity (I^2^ = 12.2%), and no evidence of inconsistency between study designs. For new‐onset AF, moderate heterogeneity was noted (I^2^ = 77.2%), but design‐specific inconsistency remained non‐significant. For RCTs, random‐effects models showed no significant treatment effects (all *p* > 0.05), and moderate heterogeneity (I^2^ = 50.5%). Cohort studies exhibited high heterogeneity (I^2^ = 74.6%). No heterogeneity was detected for follow‐up ≤ 12 months (I^2^ = 0%). For follow‐up > 12 months, high heterogeneity was observed without design inconsistency (I^2^ = 74.3%). Meta‐regression was further conducted to assess whether AF phenotype (*p* = 0.830), study design (*p* = 0.556), follow‐up duration (*p* = 0.735), or sample size (*p* = 0.839) explained the observed heterogeneity. None of the covariates were identified as significant moderators. Residual heterogeneity remained significant (all QE < 0.0001), indicating that unmeasured factors may contribute to the observed variability in treatment effects. The detailed results were shown in Table S3. Subgroup and meta‐regression analysis of network meta‐analysis, Supporting Information S1: Figure S2. Meta regression for AF type, Supporting Information S1: Figure S3. Meta regression for follow‐up duration, Supporting Information S1: Figure S4. Meta regression for sample size, Supporting Information S1: Figure S5. Meta regression for study design.

## 4 Discussion

To our knowledge, there have been network or paired meta‐analyses on the relationship between hypoglycemic drugs and AF risk. Compared with existing research, this study expands the existing evidence system through four core advantages: (1) based on 4 RCTs and 21 large‐scale cohort studies, encompassing a total of 2,171,267 T2DM patients with a larger sample size. (2) Simultaneously integrating RCTs with real world cohort studies, balancing internal validity and population extermal applicability, whereas most previous meta‐analyses only included RCT evidence. (3) Comprehensively evaluate the three clinically important endpoints of total AF, new onset AF, and AF recurrence. Previous studies have most focused on new onset AF and ignored AF recurrence outcomes. (4) Standardize GRADE evidence quality rating for all comparative outcomes, which is rarely conducted in early network meta‐analyses in this field. The adequate sample size and diverse types of evidence collectively ensure the reliability of the analytical results. The results of this network meta‐analysis indicate that SGLT‐2i significantly reduce the risk of total AF and new‐onset AF in T2DM patients compared to GLP‐1RA and DPP‐4i. This conclusion is highly consistent with current mechanistic research evidence in the field of cardiovascular metabolism, and its clinical significance also needs to be further clarified in conjunction with the limitations of existing studies. Previous guidelines have primarily focused on the glycemic control and macrovascular benefits of antidiabetic drugs, whereas the results of this study suggest that SGLT‐2i can be considered as a preferred antidiabetic regimen for patients with T2DM at high risk of AF. This finding provides new evidence‐based support for the treatment of patients with T2DM with high risk of AF. It further confirms that SGLT‐2i are not merely a “hypoglycemic agent” but a comprehensive management drug with multiple benefits, including glycemic control, macrovascular protection, and AF prevention. This has practical guiding significance for more comprehensively evaluating the value of antidiabetic drugs in clinical practice and optimizing individualized treatment strategies.

The potential protective effects of SGLT‐2i have been supported by multiple previous studies. Several meta‐analyses consistently show that compared with non‐SGLT‐2i use, SGLT‐2i can significantly reduce the risk of new‐onset AF in patients with T2DM [45, 46]. The conclusion has also been validated in a retrospective analysis based on the US Food and Drug Administration (FDA) Adverse Event Reporting System (FAERS). The results showed that the AF reporting rate among SGLT‐2i users was 4.8 per 1000 person‐years, significantly lower than that of 8.7 per 1000 person‐years among users of other hypoglycemic agents (*p* < 0.001), which supports the advantage of SGLT‐2i in AF prevention [47]. A nationwide cohort study further supplemented this evidence, revealing that SGLT‐2i could reduce AF risk by approximately 52% in T2DM patients without hypertension[aHR (95% CI): 0.48(0.25–0.91)]. However, this result should be interpreted cautiously due to the wide confidence interval of the adjusted hazard ratio [29]. A large‐sample, nationally representative cohort study using US healthcare insurance database showed that the risk of new‐onset AF in SGLT‐2i users was significantly lower than that in DPP‐4i users [48]. Another propensity score‐weighted cohort study clearly indicated that in a population with an average age of 60 years, SGLT‐2i reduced the risk of new‐onset AF by approximately 39% compared with DPP‐4i(HR (95% CI) 0.61(0.50–0.73) [33]. Animal experiments have further confirmed the mechanistic basis of that SGLT‐2i can effectively inhibit atrial remodeling, which is a core pathological process in the occurrence and maintenance of AF, explaining its clinical protective effects at the mechanistic level [49].

However, there is still controversy regarding the association between SGLT‐2i and the risk of AF. Several studies have not observed differences in the risk of AF between SGLT‐2i and placebo or other hypoglycemic agents [50]. A Nordic study in patients T2DM showed no statistically significant difference in the risk of AF between dapagliflozin users and users of new DPP‐4i after propensity score matching [51]. A cohort study conducted in Taiwan also found that the risk of AF was comparable between the SGLT‐2i treatment group and the matched control group [52]. Furthermore, previous large‐scale RCTs and meta‐analysis have also failed to reach a unified conclusion on the effect of SGLT‐2i on AF in patients with T2DM. Some studies suggest it is ineffective, while others support its potential protective role. This controversy may be related to factors such as baseline characteristics of the study population and follow‐up duration [53, 54].

Although the specific mechanism by which SGLT‐2i reduces the risk of AF are not yet fully elucidated, the AF benefits of SGLT‐2i observed in this study are essentially the results of their multi‐target direct intervention in the pathophysiological processes of AF [55, 56, 57]. First, there is a direct cardioprotective effect. SGLT‐2i can directly lower left atrial pressure and stretch stimulation, and atrial stretch is one of the core triggers of AF. This action also improves common comorbidities in patients with T2DM, further reducing upstream drivers of atrial remodeling. Previous studies have shown that empagliflozin has been proven to inhibit the expression of sodium hydrogen exchanger 1 (NHE1) in myocardial cells of patients with HF and AF, thereby alleviating adverse cardiac remodeling [58]. Second, SGLT‐2i can directly regulate myocardial electrophysiological homeostasis. In diabetic patients, upregulated myocardial NHE1 expression leads to intracellular calcium overload and increased sarcoplasmic reticulum calcium levels, which promotes AF [59]. SGLT‐2i can inhibit myocardial sodium‐hydrogen exchange, promote sodium excretion, lower intracellular calcium levels, and alleviate the proarrhythmic effects caused by calcium homeostasis imbalance [60]. In addition, SGLT‐2i can indirectly reduce AF risk by improving obesity, hypertension, hyperglycemia, and other risk factors closely associated with AF [55, 61, 62]. At the same time, epicardial fat accumulation is associated with an increased risk of AF. SGLT‐2i can reduce epicardial fat accumulation and delay myocardial fibrosis through antioxidant stress pathways [63]. This mechanism was confirmed by the study of Kang et al., showing that empagliflozin can reduce the volume of myocardial fibroblasts, alleviate extracellular matrix remodeling, and inhibit the expression of pro‐fibrotic gene markers. Subgroup analysis of the EMPA‐Hemodynamic study further showed that improvement in left atrial strain and enhancement of left atrial function were observed after 3 months of empagliflozin treatment [64]. These effects synergistically reduce adverse cardiac remodeling and myocardial hypertrophy, thereby lowering the risk of arrhythmias. However, the specific protective mechanisms of SGLT‐2i in AF‐related events still need more in‐depth research to provide a more solid theoretical basis for their clinical application.

To data, the mechanisms through which GLP‐1RA reduce the risk of AF are still under investigation. Previous cardiovascular outcome trials have clearly demonstrated that GLP‐1RA can significantly reduce the risk of MACEs, such as myocardial infarction and stroke [65, 66]. Real‐world data further validate their benefits in cardiovascular and renal risk management [67, 68]. Based on these findings, some studies recommend GLP‐1RA as first‐line therapeutic agents for patients with T2DM who have comorbid cardiovascular disease (CVD) [69]. However, it should be noted that the association between GLP‐1RA and the risk of AF has not reached a consensus, with existing evidence even exhibits contradictory results [70]. Mechanistically, GLP‐1RA may exert an indirect preventive effect against AF through pathways such as metabolic regulation, anti‐inflammatory signaling, and reduction of adverse cardiovascular events [70, 71, 72]. Conversely, GLP‐1RA may also directly act on the sinoatrial node, increasing AF risk by accelerating heart rate, thereby potentially acting as a risk factor [73]. Notably, the combination of SGLT‐2i and GLP‐1RA can synergistically improve myocardial substrate utilization, reduce inflammation and oxidative stress, promote atrial reverse remodeling, and help maintain sinus rhythm after AF ablation. Even in patients with poorly controlled HbA1c, this combined intervention can lower AF recurrence rates, highlighting that these drugs are not merely antihyperglycemic agents but also hold potential value as disease‐modifying therapies [24].

DPP‐4i represent another class of novel hypoglycemic agents for T2DM. Its core mechanism of action is to inhibit the degradation of GLP‐1, thereby increasing GLP‐1 concentration and prolonging its half‐life, which in turn stimulates insulin secretion, inhibits glycogenolysis, and ultimately regulates blood glucose [74]. Beyond glycemic control, DPP‐4i can exert anti‐inflammatory, antioxidant, and anti‐cardiac fibrotic effects through both GLP‐1 dependent and independent pathways [75]. Yamamoto et al. confirmed in a rabbit model of HF that the DPP‐4i alogliptin can alleviate atrial fibrosis and shorten the duration of AF [76]. Animal experiments further showed that DPP‐4i can improve mitochondrial function in myocardium of obese rats with insulin resistance [77]. Mitochondrial oxidative stress can lower the peak sodium current and downregulate Cx43 expression, leading to conduction abnormalities and reentrant circuit formation in atrial myocytes, ultimately promoting the occurrence and maintenance of AF [78]. The latest evidence suggests that mitochondrial dysfunction may precede structural and function changes in the atrium, forming an arrhythmogenic substrate [79], and it is speculated that mitochondrial remodeling may be the core pathway by which diabetes acts as an independent risk factor for AF [78, 80]. It is worth noting that there is a discrepancy between the protective effects of DPP‐4i on cardiac remodeling observed in basic research and their overall clinical benefits. Therefore, the specific mechanisms by which DPP‐4i modulate AF‐related risk in patients with T2DM still need to be further clarified.

In summary, the results of this study provide important supplementation and evidence‐based support to the existing evidence. The benefit of SGLT‐2i in reducing AF observed in this study is essentially due to its multi‐target direct intervention in the pathophysiological process of AF. In contrast, GLP‐1RA and DPP‐4i did not demonstrate superior AF benefits compared to SGLT‐2i, which is highly related to the indirect nature of their mechanisms of action. Both classes of drugs primarily focus on improving metabolic disturbances and lack direct intervention targets for atrial remodeling, hence their effects on AF prevention are at a comparable level. Furthermore, no significant differences were observed among the three drug classes in their impact on AF recurrence risk, primarily due to the irreversibility of atrial structural remodeling. Once AF occurs, electrical and structural remodeling of the atrium enters a progressive phase. The advantage of SGLT‐2i lies in blocking the early remodeling process of newly developed AF, but it has limited efficacy in intervening with established pathological remodeling lesions. This suggests that the AF benefits of SGLT‐2i are more applicable to primary prevention in high‐risk populations who have not yet developed AF. For patients already diagnosed with AF, traditional antiarrhythmic therapy should remain the core treatment, with SGLT‐2i serving only as an adjuvant for synergistic management. This conclusion provides an important cardiovascular‐metabolic perspective for the development of individualized glucose‐lowering regimens in patients with T2DM. Glucose‐lowering therapy is no longer limited to glycemic control but should also involve selecting drugs with targeted protective effects based on AF risk stratification. However, large‐scale clinical trials with AF‐related outcomes as primary endpoints are still relatively scarce. The results of this study should be considered exploratory, and their reliability and generalizability need to be further validated through future large‐scale, multi‐center RCTs to provide more robust evidence‐based support for clinical decision‐making.

### 4.1 Limitations

This study also has certain limitations. First, a relatively high proportion of cohort studies were included, and the observational design makes it difficult to completely avoid interference from confounding factors, such as unevenness in variables like patients' underlying heart disease and concomitant medications. Although sensitivity analysis verified the robustness of the results, the lack of high‐quality RCT evidence may still weaken the argument strength of the conclusions. Second, although we attempted to retrieve unpublished studies to reduce publication bias, the inherent risk of bias where positive results are more likely to be published cannot be completely eliminated. This bias may potentially affect the estimation of the pooled effect size, leading to an overestimation or underestimation of the true benefits of the drug. Third, after excluding small‐sample studies, the effect of SGLT‐2i on reducing the risk of AF recurrence changed from “a trend toward reduction” to “statistically significant reduction”. This suggests that the heterogeneity of small‐sample studies may have diluted the true efficacy of SGLT‐2i, thereby affecting the robustness of the results for this outcome. Fourth, there is a lack of unified diagnostic criteria for AF across different studies, and missed diagnosis of asymptomatic AF may lead to result bias. Fifth, this study did not further analyze the effect differences of different subtypes of SGLT‐2i and different dosages, so it cannot provide more precise medication guidance for clinical practice. Sixth, as a network meta‐analysis, this study can only reveal the association between SGLT‐2i, GLP‐1RA, DPP‐4i, and AF related outcomes, rather than confirming a causal relationships. Based on the above limitations, future studies need to further conduct high‐quality RCTs with AF as the primary outcome endpoint to clarify the efficacy differences of SGLT‐2i in different populations. At the same time, explore the combined application value of SGLT‐2i and antiarrhythmic drugs to provide more comprehensive evidence‐based support for the comprehensive management of AF in patients with T2DM.

## 5 Conclusion

The management of T2DM involves the prevention of subsequent cardiovascular complications. The results of this network meta‐analysis indicate that SGLT2i is associated with a lower risk of AF in T2DM patients compared to GLP‐1RA and DPP‐4i. Sensitivity analysis further confirms that SGLT‐2i significantly reduces the risk of AF recurrence statistically. These finding suggests that SGLT‐2i should be considered as a preferred antidiabetic regimen for patients with T2DM at high risk of AF.

## Author Contributions

X.J. Ye and S.H. Wang planed and designed the research. Q. Wu, L.Y. Hou, and X.Z. Hou provided methodological support. Q. Wu, Y.T. Yang and C.Y. Yang extract data. X.J. Ye, and X.Z. Hou performed the statistical analysis. X.J. Ye wrote the manuscript. S.H. Wang reviewed and revised the initial manuscript. All authors reviewed and approved the final version of the manuscript.

## Funding

This work was supported by National Natural Science Foundation of China (82374421), Chinese Academy of Traditional Chinese Medicine's Major Tackling Project of Science and Technology Innovation (CI2021A00921), Beijing Municipal Natural Science Foundation (7232311).

## Consent

The authors have nothing to report.

## Conflicts of Interest

The authors declare no conflicts of interest.

## Supporting information


Supporting Information S1

**Table S1:** Transitivity testing results for network meta‐analysis.

**Table S2:** Inconsistency assessment by node splitting method.

**Table S3:** Subgroup and meta‐regression analysis of network meta‐analysis.

**Table S4:** System retrieval strategy for MEDLINE.

**Table S5:** System retrieval strategy for Cochrane Central Register of Controlled Trials (CENTRAL).

**Table S6:** System retrieval strategy for Embase.

**Table S7:** System retrieval strategy for Clinical Trials.gov.

## Data Availability

Data sharing not applicable to this article as no datasets were generated or analysed during the current study.
