# Supplementary material for: Network and Pairwise Meta‐Analysis of the Association Between Novel Hypoglycemic Agents and Atrial Fibrillation Risk in Patients With Type 2 Diabetes Mellitus
Source: Diabetes Metab Res Rev. 2026 Jul 15;42(5):e70202. doi: 10.1002/dmrr.70202 (PMC13372237; doi:10.1002/dmrr.70202)
Supplement: Supplementary file 1 — Supporting Information S1 [file DMRR-42-e70202-s002.docx]

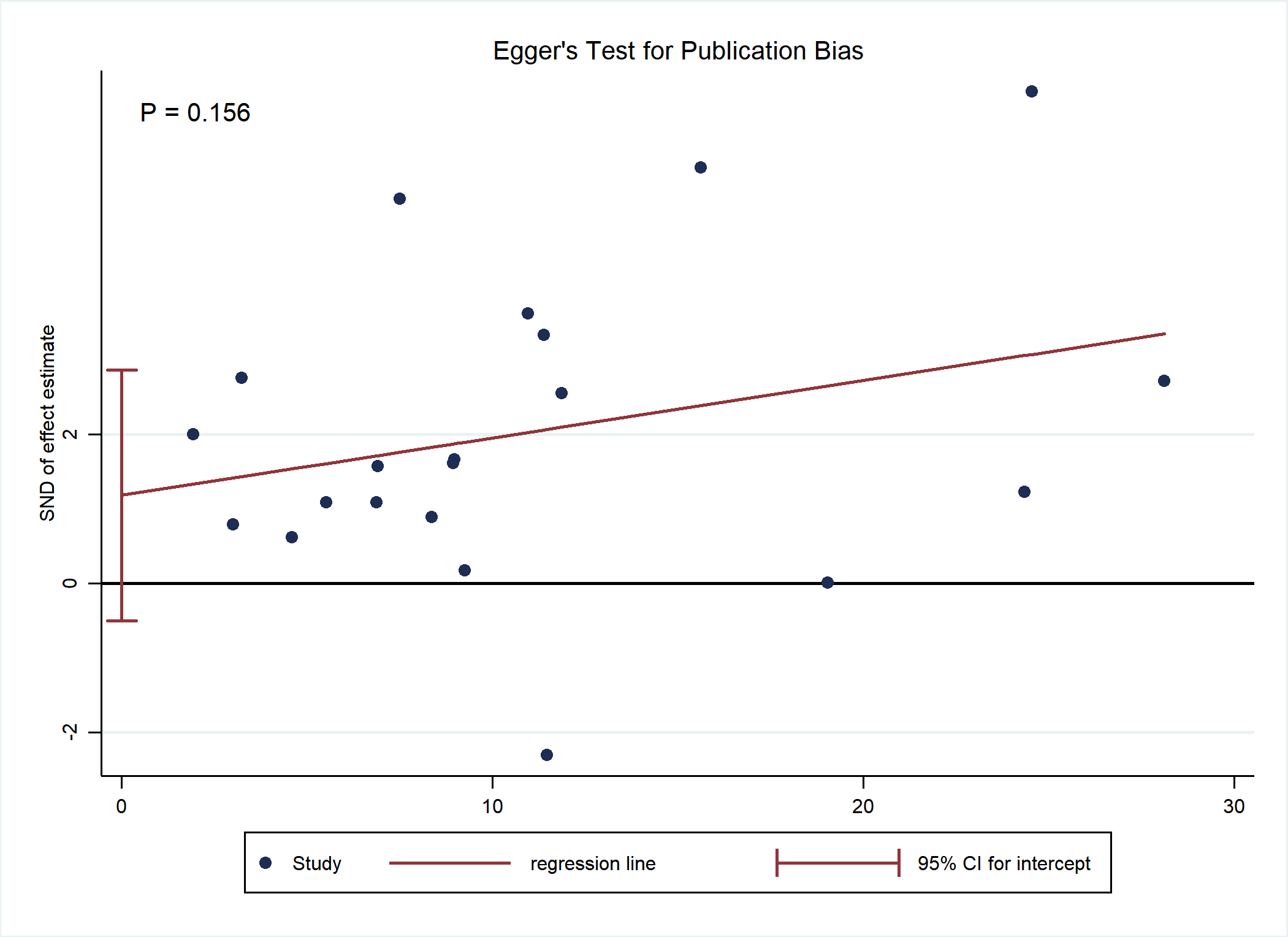


**Supplementary Figure S1. Egger's regression test for Publication Bias.**

**
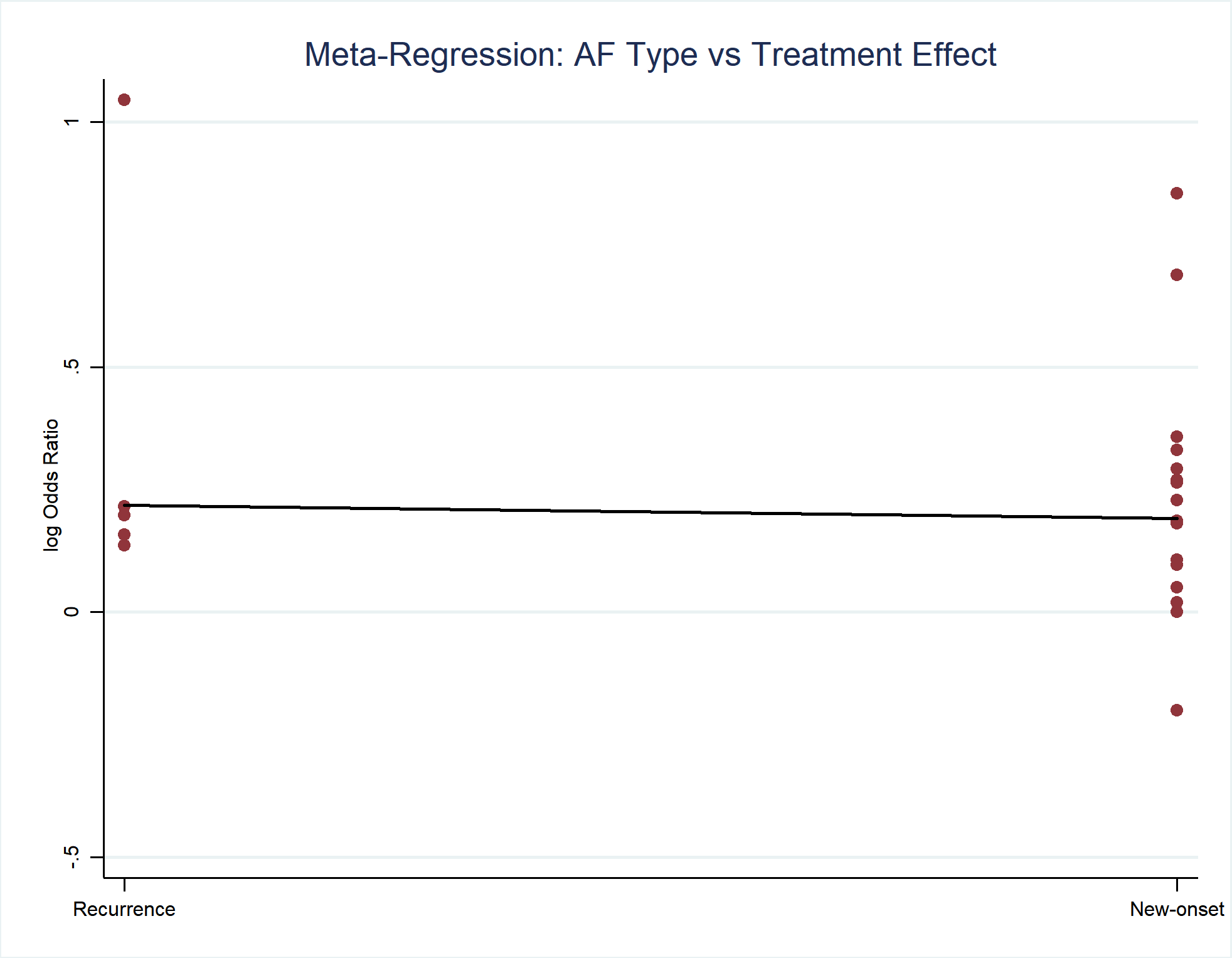
**

**Supplementary Figure S2. Meta regression for AF type.**

**
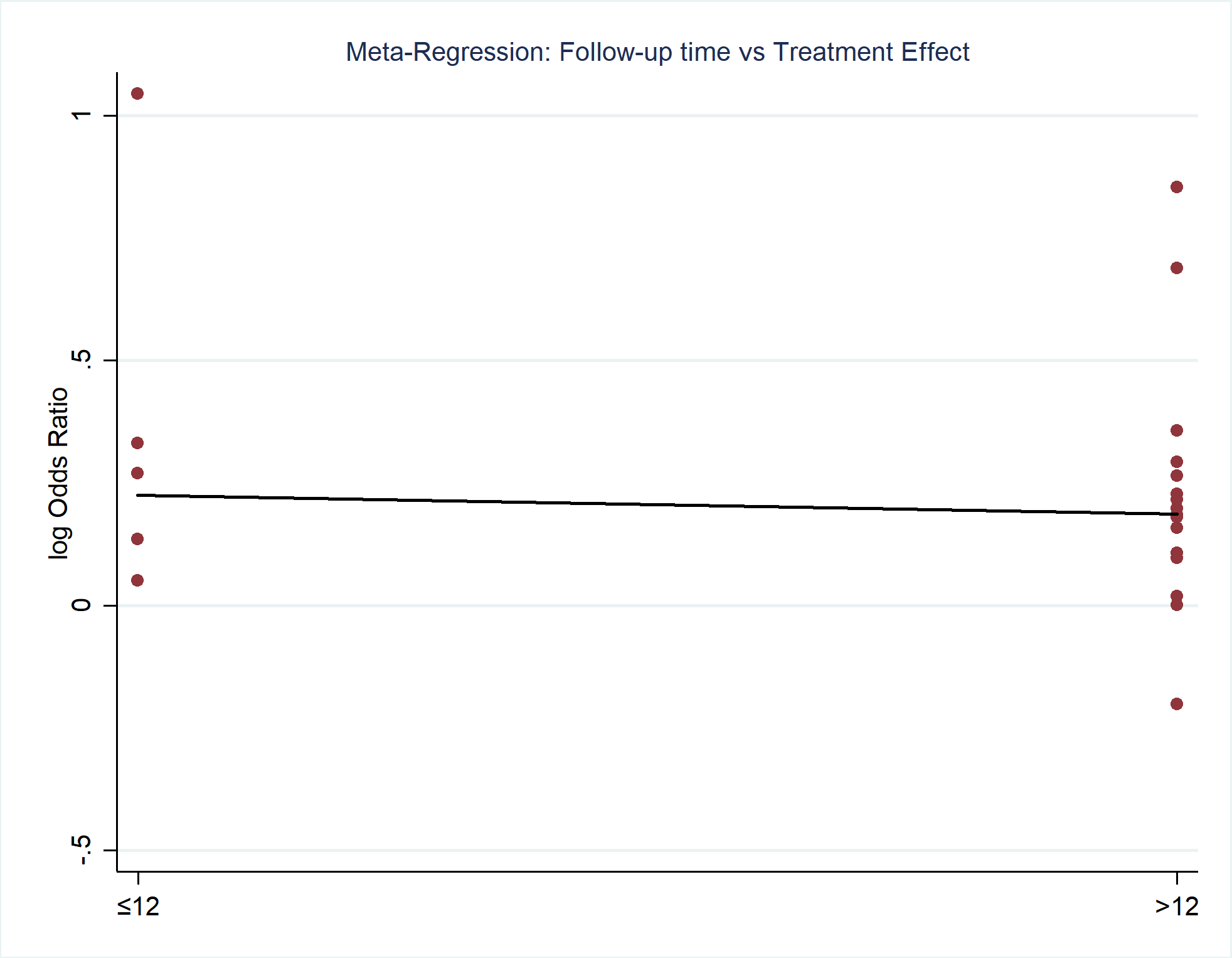
**

**Supplementary Figure S3. Meta regression for follow-up duration.**

**
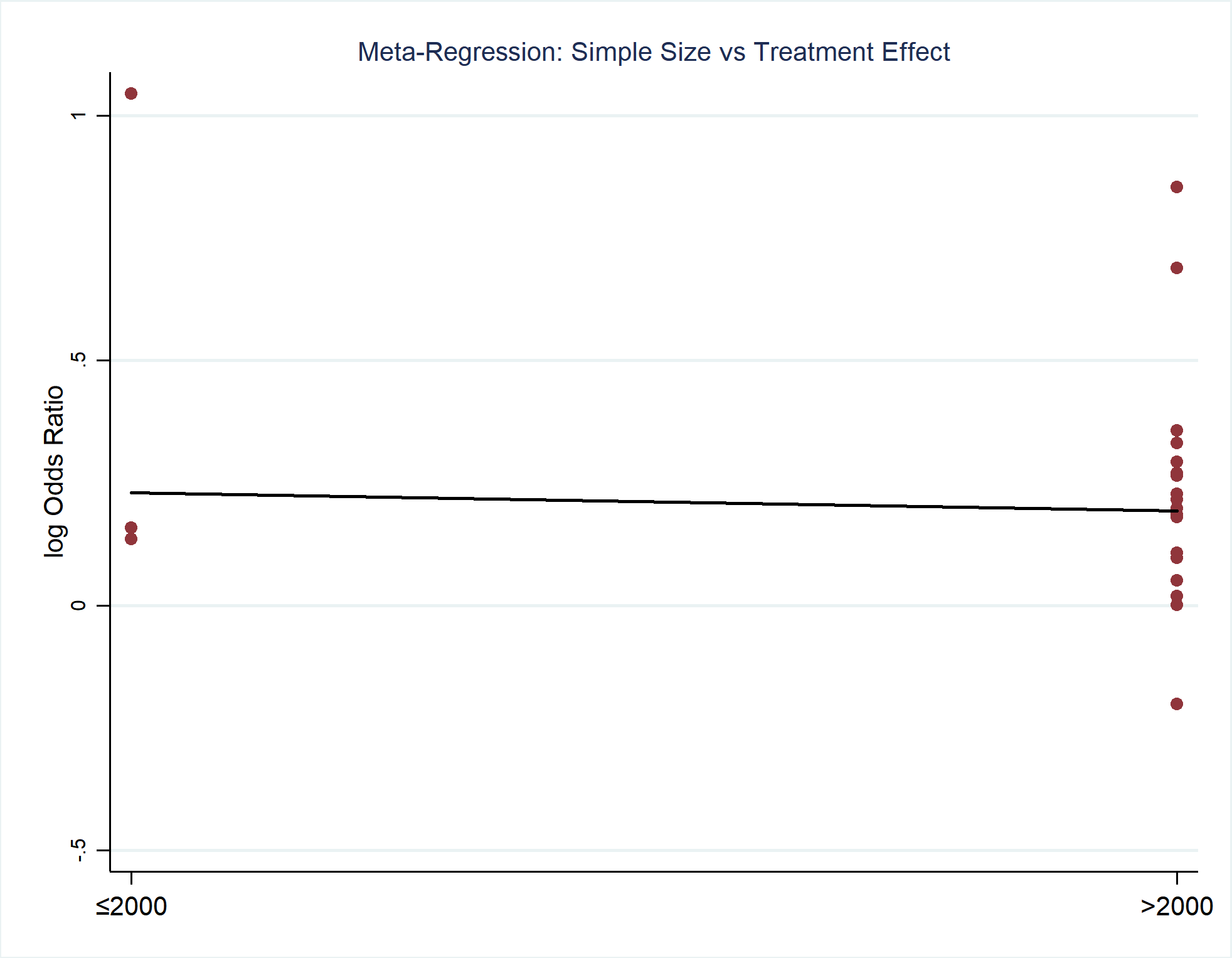
**

**Supplementary Figure S4. Meta regression for sample size.**

**
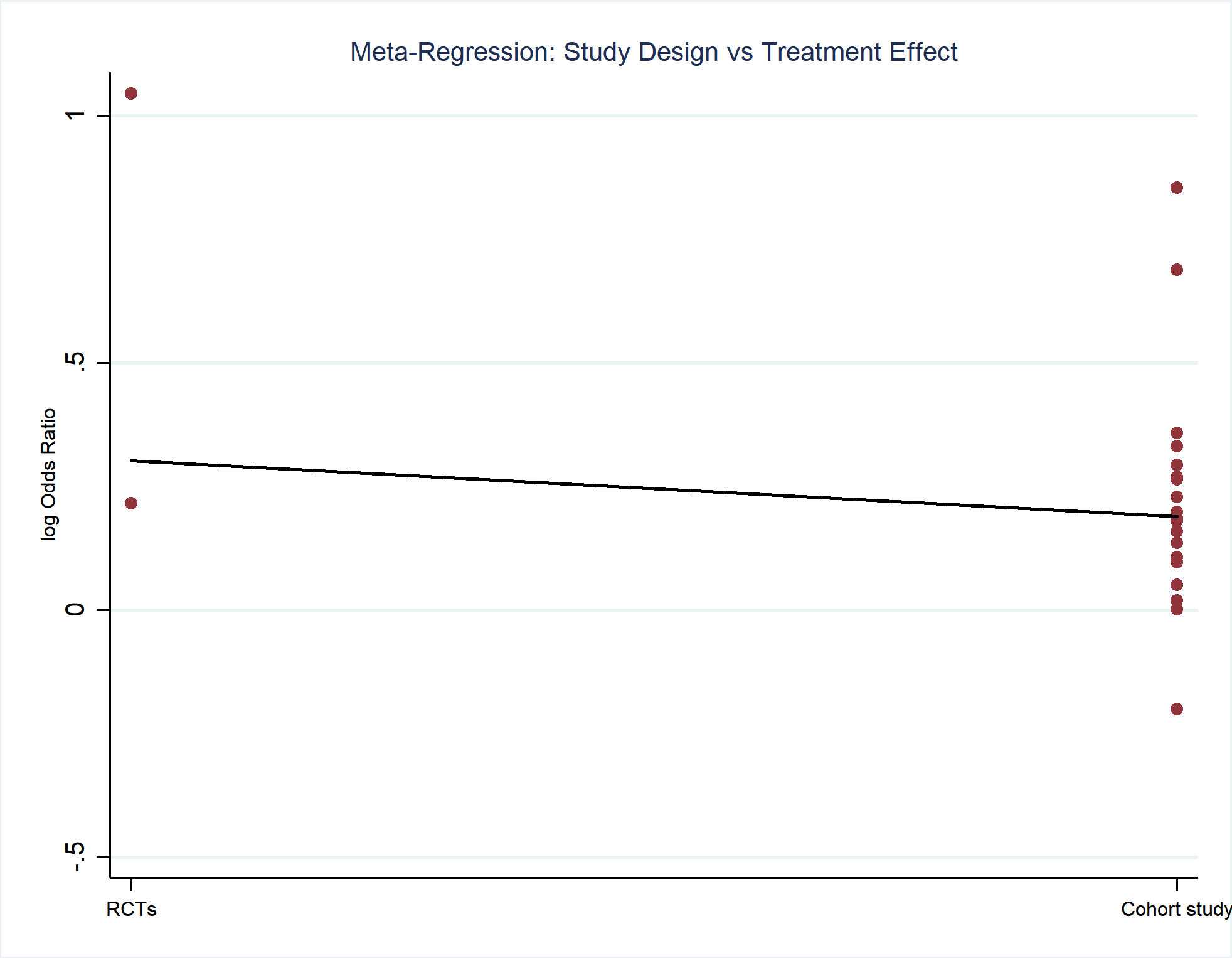
**

**Supplementary Figure S5. Meta regression for study design.**
