# Supplementary material for: Network and Pairwise Meta‐Analysis of the Association Between Novel Hypoglycemic Agents and Atrial Fibrillation Risk in Patients With Type 2 Diabetes Mellitus
Source: Diabetes Metab Res Rev. 2026 Jul 15;42(5):e70202. doi: 10.1002/dmrr.70202 (PMC13372237; doi:10.1002/dmrr.70202)
Supplement: Supplementary file 2 — Table S1: Transitivity testing results for network meta‐analysis. [file DMRR-42-e70202-s008.docx]

Supplementary table S1. Transitivity testing results for network meta-analysis

| **Covariates** | **Sample size** | **Follow-up duration** | **Male proportion** |
| --- | --- | --- | --- |
| P | 0.4574 | 0.5149 | 0.3258 |
