# Supplementary material for: Network and Pairwise Meta‐Analysis of the Association Between Novel Hypoglycemic Agents and Atrial Fibrillation Risk in Patients With Type 2 Diabetes Mellitus
Source: Diabetes Metab Res Rev. 2026 Jul 15;42(5):e70202. doi: 10.1002/dmrr.70202 (PMC13372237; doi:10.1002/dmrr.70202)
Supplement: Supplementary file 3 — Table S2: Inconsistency assessment by node splitting method. [file DMRR-42-e70202-s004.docx]

Supplementary table S2. Inconsistency assessment by node splitting method

| **Side** | **Direct** | | **Indirect** | | **Difference** | |  |
| --- | --- | --- | --- | --- | --- | --- | --- |
|  | **Coef.** | **Std. Err.** | **Coef.** | **Std. Err.** | **Coef.** | **Std. Err.** | **P** |
| SGLT-2i - GLP-1RA | .1545444 | .0734137 | .1676315 | .0970694 | -.013087 | .1212333 | 0.914 |
| SGLT-2i - DPP-4i | .2284364 | .0559609 | .2221271 | .1168669 | .0063093 | .1290206 | 0.961 |
| SGLT-2i - placebo | .1247242 | .1246478 | .0996332 | .1834385 | .025091 | .2219126 | 0.910 |
| GLP-1RA - DPP-4i | .064571 | .0939294 | .0708871 | .0884134 | -.0063161 | .1290171 | 0.961 |
| GLP-1RA - placebo | -.0576552 | .1729886 | -.0325325 | .1389904 | -.0251227 | .2219087 | 0.910 |
