# Supplementary material for: Network and Pairwise Meta‐Analysis of the Association Between Novel Hypoglycemic Agents and Atrial Fibrillation Risk in Patients With Type 2 Diabetes Mellitus
Source: Diabetes Metab Res Rev. 2026 Jul 15;42(5):e70202. doi: 10.1002/dmrr.70202 (PMC13372237; doi:10.1002/dmrr.70202)
Supplement: Supplementary file 4 — Table S3: Subgroup and meta‐regression analysis of network meta‐analysis. [file DMRR-42-e70202-s007.docx]

Supplementary table S3. Subgroup and meta-regression analysis of network meta-analysis

| **Covariates** | **Subgroup** | **Heterogeneity** | | **Meta-regression** |
| --- | --- | --- | --- | --- |
|  |  | **I^2^** | **Q** |  |
| AF phenotype | Recurrent AF | 12.2% | 2.28 | 0.830 |
|  | New-onset AF | 77.2% | 74.53 |  |
| Study design | RCTs | 50.5% | 2.02 | 0.918 |
|  | Cohort studies | 74.6% | 74.8 |  |
| Follow-up duration | ≤12 months | 0% | 1.86 | 0.3 |
|  | >12 months | 74.3% | 66.17 |  |
| Sample size | - | - | - | 0.096 |
|  | - | - | - |  |
