# Supplementary material for: Network and Pairwise Meta‐Analysis of the Association Between Novel Hypoglycemic Agents and Atrial Fibrillation Risk in Patients With Type 2 Diabetes Mellitus
Source: Diabetes Metab Res Rev. 2026 Jul 15;42(5):e70202. doi: 10.1002/dmrr.70202 (PMC13372237; doi:10.1002/dmrr.70202)
Supplement: Supplementary file 5 — Table S4: System retrieval strategy for MEDLINE. [file DMRR-42-e70202-s003.docx]

Supplementary Table S4. System retrieval strategy for MEDLINE

| Supplementary Table S4. System retrieval strategy |
| --- |
| #1 ("Sodium-Glucose Transporter 2 Inhibitors"[MeSH Terms] OR "SGLT-2 inhibitors"[Title/Abstract] OR "SGLT-2i"[Title/Abstract] OR "Canagliflozin"[Title/Abstract] OR "Dapagliflozin"[Title/Abstract] OR "Empagliflozin"[Title/Abstract] OR "Ertugliflozin"[Title/Abstract] OR "Ipragliflozin"[Title/Abstract] OR "Luseogliflozin"[Title/Abstract]) |
| #2 ("Dipeptidyl Peptidase-4 Inhibitors"[MeSH Terms] OR "DPP-4 inhibitors"[Title/Abstract] OR "DPP-4i"[Title/Abstract] OR "Sitagliptin"[Title/Abstract] OR "Saxagliptin"[Title/Abstract] OR "Linagliptin"[Title/Abstract] OR "Alogliptin"[Title/Abstract] OR "Vildagliptin"[Title/Abstract] OR "Voglibose"[Title/Abstract]) |
| #3 ("Glucagon-Like Peptide-1 Receptor Agonists"[MeSH Terms] OR "GLP-1 receptor agonist"[Title/Abstract] OR "GLP-1RA"[Title/Abstract] OR "Exenatide"[Title/Abstract] OR "Liraglutide"[Title/Abstract] OR "Dulaglutide"[Title/Abstract] OR "Tirzepatide"[Title/Abstract] OR "Semaglutide"[Title/Abstract] OR "Lixisenatide"[Title/Abstract]) |
| #4 ("Atrial Fibrillation"[MeSH Terms] OR "atrial fibrillation"[Title/Abstract] OR "AF"[Title/Abstract]) |
| #5 ("Randomized Controlled Trial"[pt] OR "Controlled Clinical Trial"[pt] OR "cohort study"[pt] OR "prospective study"[Title/Abstract] OR "retrospective study"[Title/Abstract]) |
| #6 #1 OR #2 OR #3 AND #4 AND #5 |
