# Supplementary material for: Network and Pairwise Meta‐Analysis of the Association Between Novel Hypoglycemic Agents and Atrial Fibrillation Risk in Patients With Type 2 Diabetes Mellitus
Source: Diabetes Metab Res Rev. 2026 Jul 15;42(5):e70202. doi: 10.1002/dmrr.70202 (PMC13372237; doi:10.1002/dmrr.70202)
Supplement: Supplementary file 6 — Table S5: System retrieval strategy for Cochrane Central Register of Controlled Trials (CENTRAL). [file DMRR-42-e70202-s006.docx]

Supplementary Table S5. System retrieval strategy for Cochrane Central Register of Controlled Trials (CENTRAL)

| Supplementary Table S5. System retrieval strategy for CENTRAL |
| --- |
| #1 MeSH descriptor: [Sodium-Glucose Transporter 2 Inhibitors] explode all trees |
| #2 "SGLT-2 inhibitors" OR "SGLT-2i" OR "Canagliflozin" OR "Dapagliflozin" OR "Empagliflozin" OR "Ertugliflozin" OR "Ipragliflozin" OR "Luseogliflozin" |
| #3 (#1 OR #2) |
| #4 MeSH descriptor: [Dipeptidyl Peptidase-4 Inhibitors] explode all trees |
| #5 "DPP-4 inhibitors" OR "DPP-4i" OR "Sitagliptin" OR "Saxagliptin" OR "Linagliptin" OR "Alogliptin" OR "Vildagliptin" OR "Voglibose" |
| #6 (#4 OR #5) |
| #7 MeSH descriptor: [Glucagon-Like Peptide-1 Receptor Agonists] explode all trees |
| #8 "GLP-1 receptor agonist" OR "GLP-1RA" OR "Exenatide" OR "Liraglutide" OR "Dulaglutide" OR "Tirzepatide" OR "Semaglutide" OR "Lixisenatide" |
| #9 (#7 OR #8) |
| #10 MeSH descriptor: [Atrial Fibrillation] explode all trees |
| #11 "atrial fibrillation" OR "AF" |
| #12 #10 OR #11 |
| #13 #3 OR #6 OR #9 AND #12 |
