# Supplementary material for: Network and Pairwise Meta‐Analysis of the Association Between Novel Hypoglycemic Agents and Atrial Fibrillation Risk in Patients With Type 2 Diabetes Mellitus
Source: Diabetes Metab Res Rev. 2026 Jul 15;42(5):e70202. doi: 10.1002/dmrr.70202 (PMC13372237; doi:10.1002/dmrr.70202)
Supplement: Supplementary file 7 — Table S6: System retrieval strategy for Embase. [file DMRR-42-e70202-s005.docx]

Supplementary Table S6. System retrieval strategy for Embase

| Supplementary Table S6. System retrieval strategy for Embase. |
| --- |
| #1 'sodium glucose transporter 2 inhibitor'/exp OR 'sglt-2 inhibitor':ab,ti OR 'sglt-2i':ab,ti OR 'canagliflozin':ab,ti OR 'dapagliflozin':ab,ti OR 'empagliflozin':ab,ti OR 'ertugliflozin':ab,ti OR 'ipragliflozin':ab,ti OR 'luseogliflozin':ab,ti |
| #2 'dipeptidyl peptidase-4 inhibitor'/exp OR 'dpp-4 inhibitor':ab,ti OR 'dpp-4i':ab,ti OR 'sitagliptin':ab,ti OR 'saxagliptin':ab,ti OR 'linagliptin':ab,ti OR 'alogliptin':ab,ti OR 'vildagliptin':ab,ti OR 'voglibose':ab,ti |
| #3 'glucagon-like peptide-1 receptor agonist'/exp OR 'glp-1 receptor agonist':ab,ti OR 'glp-1ra':ab,ti OR 'exenatide':ab,ti OR 'liraglutide':ab,ti OR 'dulaglutide':ab,ti OR 'tirzepatide':ab,ti OR 'semaglutide':ab,ti OR 'lixisenatide':ab,ti |
| #4 'atrial fibrillation'/exp OR 'atrial fibrillation':ab,ti OR 'af':ab,ti |
| #5 'randomized controlled trial'/exp OR 'controlled clinical trial'/exp OR 'cohort study'/exp OR 'prospective study':ab,ti OR 'retrospective study':ab,ti |
| #6 (#1 OR #2 OR #3) AND #4 AND #5 |
