# Supplementary material for: Network and Pairwise Meta‐Analysis of the Association Between Novel Hypoglycemic Agents and Atrial Fibrillation Risk in Patients With Type 2 Diabetes Mellitus
Source: Diabetes Metab Res Rev. 2026 Jul 15;42(5):e70202. doi: 10.1002/dmrr.70202 (PMC13372237; doi:10.1002/dmrr.70202)
Supplement: Supplementary file 8 — Table S7: System retrieval strategy for Clinical Trials.gov. [file DMRR-42-e70202-s001.docx]

Supplementary Table S7. System retrieval strategy for Clinical Trials.gov

| Supplementary Table S7. System retrieval strategy for Clinical Trials.gov. |
| --- |
| Intervention:  Sodium-Glucose Transporter 2 Inhibitors OR SGLT-2 inhibitors OR SGLT-2i OR Canagliflozin OR Dapagliflozin OR Empagliflozin OR Ertugliflozin OR Ipragliflozin OR Luseogliflozin  OR  Dipeptidyl Peptidase-4 Inhibitors OR DPP-4 inhibitors OR DPP-4i OR Sitagliptin OR Saxagliptin OR Linagliptin OR Alogliptin OR Vildagliptin OR Voglibose  OR  Glucagon-Like Peptide-1 Receptor Agonists OR GLP-1 receptor agonist OR GLP-1RA OR Exenatide OR Liraglutide OR Dulaglutide OR Tirzepatide OR Semaglutide OR Lixisenatide |
| Condition:  Atrial Fibrillation OR AF |
| Study Type:  Interventional (Clinical Trial) OR Observational (Cohort) |
